# Supplementary material for: Evaluation of the Kinetics of Pancreatic Stone Protein as a Predictor of Ventilator-Associated Pneumonia
Source: Biomedicines. 2023 Sep 29;11(10):2676. doi: 10.3390/biomedicines11102676 (PMC10604889; doi:10.3390/biomedicines11102676)
Supplement: Supplementary file 1 [file biomedicines-11-02676-s001.zip › biomedicines-2609536-supplementary.pdf]

Evaluation of the kinetics of Pancreatic Stone Protein as a Predictor of Ventilator-Associated Pneumonia. A post-hoc analysis of BioVAP Study.

Supplementary

Table S1. Posterior summaries of the more relevant parameters and hyperparameters of the joint model 1.

	Mean	SD	2.5%	97.5%	P
**Survival process (VAP)**					
PSP (normalized to 0-1 range) value	-0.0113	0.3851	-0.7754	0.7501	0.9649
PSP (normalized to 0-1 range) daily change	-0.1778	1.1693	-2.5444	2.0648	0.8981
PSP (normalized to 0-1 range) cumulative area	0.0425	0.3929	-0.7331	0.8196	0.9009
Infected (vs. Non-infected)	-1.0638	0.5657	-2.2247	-0.0326	0.0431
**Longitudinal process for PSP (normalized to 0-1 range)**					
Intercept	-2.4376	0.6158	-3.6643	-1.2383	0.0002
N_1(days)	0.3514	1.1466	-1.9007	2.5830	0.7543
N_2(days)	-0.4077	0.6505	-1.7131	0.8473	0.5211
Infected (vs. Non-infected)	0.4618	0.3856	-0.2989	1.2182	0.2341
Apache II	0.0506	0.0160	0.0193	0.0825	0.0011
CRP	0.0309	0.0177	-0.0033	0.0659	0.0765
σ	4.8339	0.4396	3.9792	5.7036	<0.0001

The terms N_i(days) denote the basis for a natural cubic spline with 2 degrees of freedom.

P, tail probability [2×min{P(·>0),P(·<0)}]; PSP, pancreatic stone protein; SD, standard deviation; VAP, ventilator-associated pneumonia.

Table S2. Posterior summaries of the more relevant parameters and hyperparameters of the joint model 2.

	Mean	SD	2.5%	97.5%	P
**Survival process (VAP)**					
PSP (normalized to 0-1 range) value	0.0705	0.1236	-0.1729	0.3128	0.5636
log(CRP) value	-0.0009	0.1414	-0.2802	0.2857	0.9765
log(PCT) value	-0.0855	0.0944	-0.2784	0.0959	0.3510
Infected (vs. Non-infected)	-1.0327	0.5528	-2.1590	-0.0091	0.0484
**Longitudinal process for PSP (normalized to 0-1 range)**					
Intercept	-2.7043	0.7220	-4.1448	-1.2958	0.0001
N_1(days)	0.4515	0.5943	-0.7035	1.6011	0.4440
N_2(days)	0.2083	1.5339	-2.7473	3.2352	0.8901
N_3(days)	-0.4421	1.1345	-2.7160	1.7410	0.6944
Infected (vs. Non-infected)	0.4981	0.4123	-0.3123	1.3184	0.2277
Apache II	0.0535	0.0171	0.0199	0.0868	0.0030
CRP	0.0412	0.0211	-0.0002	0.0831	0.0509
σ	5.5279	0.5040	4.5595	6.5342	<0.0001
**Longitudinal process for log(CRP)**					
Intercept	1.1246	0.2520	0.6366	1.6243	<0.0001
N_1(days)	-0.2891	0.6744	-1.6260	1.0344	0.6601
N_2(days)	0.4742	0.7043	-0.8888	1.9017	0.4933
N_3(days)	-0.2065	0.7108	-1.5968	1.2052	0.7698
Infected (vs. Non-infected)	-0.0740	0.1367	-0.3421	0.1931	0.5893
Apache II	0.0010	0.0059	-0.0104	0.0126	0.8522
CRP	0.0700	0.0071	0.0562	0.0842	<0.0001
σ	0.4544	0.0401	0.3977	0.5524	<0.0001
**Longitudinal process for log(PCT)**					
Intercept	-2.2240	0.4880	-3.1840	-1.2634	<0.0001
N_1(days)	-2.5195	1.2760	-5.0520	-0.0087	0.0495
N_2(days)	0.3525	1.0732	-1.7270	2.4686	0.7478
N_3(days)	0.5202	0.9883	-1.4281	2.4699	0.5914

	Mean	SD	2.5%	97.5%	P
Infected (vs. Non-infected)	1.1243	0.2819	0.5747	1.6779	0.0005
Apache II	0.0503	0.0124	0.0259	0.0750	<0.0001
CRP	0.0592	0.0140	0.0323	0.0868	0.0001
σ	0.8884	0.0692	0.7919	1.0552	<0.0001

The terms N_i(days) denote the basis for a natural cubic spline with 3 degrees of freedom.
CRP, C-reactive protein; P, tail probability [2×min{P(·>0),P(·<0)}]; PCT, procalcitonin; PSP, pancreatic stone protein; SD, standard deviation; VAP, ventilator-associated pneumonia.

Table S3. Posterior summaries of the more relevant parameters and hyperparameters of the joint model 3.

	Mean	SD	2.5%	97.5%	P
**Survival process (VAP)**					
PSP (normalized to 0-1 range) daily change	0.7897	0.6091	-0.4067	2.0085	0.1884
log(CRP) daily change	-0.3548	0.5351	-1.4259	0.6670	0.5090
log(PCT) daily change	-0.4270	0.4359	-1.3056	0.4548	0.2979
Infected (vs. Non-infected)	-1.0420	0.5599	-2.1925	-0.0273	0.0417
**Longitudinal process for PSP (normalized to 0-1 range)**					
Intercept	-2.6909	0.7204	-4.1400	-1.2891	0.0001
N_1(days)	0.4695	0.5878	-0.6733	1.6403	0.4207
N_2(days)	0.1392	1.5120	-2.7917	3.1393	0.9316
N_3(days)	-0.4724	1.1292	-2.7291	1.6850	0.6721
Infected (vs. Non-infected)	0.5094	0.4145	-0.3069	1.3229	0.2143
Apache II	0.0534	0.0170	0.0204	0.0874	0.0022
CRP	0.0414	0.0211	0.0004	0.0832	0.0480
σ	5.5677	0.5142	4.5852	6.5960	<0.0001
**Longitudinal process for log(CRP)**					
Intercept	1.1211	0.2502	0.6377	1.6203	<0.0001
N_1(days)	-0.2954	0.6789	-1.6066	1.0586	0.6502
N_2(days)	0.4777	0.7050	-0.9073	1.8777	0.4938
N_3(days)	-0.2060	0.7107	-1.5896	1.1825	0.7668
Infected (vs. Non-infected)	-0.0723	0.1375	-0.3397	0.1963	0.6009
Apache II	0.0011	0.0058	-0.0102	0.0127	0.8395
CRP	0.0700	0.0071	0.0563	0.0843	<0.0001
σ	0.4547	0.0406	0.3980	0.5547	<0.0001
**Longitudinal process for log(PCT)**					
Intercept	-2.2255	0.4851	-3.1841	-1.2722	<0.0001
N_1(days)	-2.5026	1.2748	-5.0330	0.0579	0.0559
N_2(days)	0.3436	1.0734	-1.7958	2.4521	0.7449
N_3(days)	0.5081	0.9853	-1.4300	2.4724	0.5963

	Mean	SD	2.5%	97.5%	P
Infected (vs. Non-infected)	1.1170	0.2841	0.5690	1.6703	0.0005
Apache II	0.0502	0.0124	0.0259	0.0748	<0.0001
CRP	0.0596	0.0139	0.0325	0.0867	<0.0001
σ	0.8885	0.0686	0.7931	1.0585	<0.0001

The terms N_i(days) denote the basis for a natural cubic spline with 3 degrees of freedom.
CRP, C-reactive protein; P, tail probability [2×min{P(·>0),P(·<0)}]; PCT, procalcitonin; PSP, pancreatic stone protein; SD, standard deviation; VAP, ventilator-associated pneumonia.

Table S4. Posterior summaries of the more relevant parameters and hyperparameters of the joint model 4.

	Mean	SD	2.5%	97.5%	P
**Survival process (VAP)**					
PSP (normalized to 0-1 range) value	0.0512	0.1284	-0.1992	0.3053	0.6857
PSP (normalized to 0-1 range) daily change	0.8170	0.5902	-0.3403	1.9991	0.1522
log(CRP) value	0.0051	0.1423	-0.2721	0.2881	0.9741
log(CRP) daily change	-0.3955	0.5528	-1.5180	0.6613	0.4744
log(PCT) value	-0.0729	0.0998	-0.2840	0.1144	0.4620
log(PCT) daily change	-0.3792	0.4592	-1.3204	0.5350	0.3733
Infected (vs. Non-infected)	-1.0386	0.5604	-2.1924	-0.0085	0.0479
**Longitudinal process for PSP (normalized to 0-1 range)**					
Intercept	-2.7163	0.7230	-4.1716	-1.3288	<0.0001
N_1(days)	0.4630	0.5936	-0.6803	1.6256	0.4340
N_2(days)	0.2092	1.4925	-2.7112	3.1962	0.9031
N_3(days)	-0.4199	1.1068	-2.7088	1.7489	0.7032
Infected (vs. Non-infected)	0.5184	0.4141	-0.2838	1.3314	0.2099
Apache II	0.0537	0.0171	0.0200	0.0876	0.0022
CRP	0.0412	0.0206	0.0010	0.0818	0.0448
σ	5.5601	0.5150	4.5787	6.5920	<0.0001
**Longitudinal process for log(CRP)**					
Intercept	1.1213	0.2498	0.6338	1.6101	0.0001
N_1(days)	-0.2857	0.6800	-1.6141	1.0488	0.6689
N_2(days)	0.4720	0.7093	-0.9294	1.8712	0.4999
N_3(days)	-0.2152	0.7192	-1.6155	1.1891	0.7544
Infected (vs. Non-infected)	-0.0726	0.1362	-0.3421	0.1936	0.5958
Apache II	0.0011	0.0058	-0.0103	0.0126	0.8568
CRP	0.0701	0.0071	0.0560	0.0843	<0.0001
σ	0.4545	0.0404	0.3979	0.5534	<0.0001
**Longitudinal process for log(PCT)**					
Intercept	-2.2212	0.4859	-3.1656	-1.2535	<0.0001

	Mean	SD	2.5%	97.5%	P
N_1(days)	-2.5231	1.2558	-4.9786	-0.0328	0.0474
N_2(days)	0.3468	1.0678	-1.7540	2.4805	0.7427
N_3(days)	0.5168	0.9773	-1.3907	2.4386	0.5964
Infected (vs. Non-infected)	1.1149	0.2834	0.5592	1.6675	0.0002
Apache II	0.0502	0.0124	0.0260	0.0748	0.0001
CRP	0.0594	0.0139	0.0324	0.0876	<0.0001
σ	0.8874	0.0669	0.7923	1.0546	<0.0001

The terms N_i(days) denote the basis for a natural cubic spline with 3 degrees of freedom.

C-RP, C-reactive protein; P, tail probability [2×min{P(·>0),P(·<0)}]; PCT, procalcitonin; PSP, pancreatic stone protein; SD, standard deviation; VAP, ventilator-associated pneumonia.

Table S5. Posterior summaries of the more relevant parameters and hyperparameters of the joint model 5, including only patients with non-infection at admission.

	Mean	SD	2.5%	97.5%	P
**Survival process (VAP)**					
PSP (normalized to 0-1 range) value	0.3672	0.3626	-0.3121	1.1216	0.2935
PSP (normalized to 0-1 range) daily change	-0.4091	0.9536	-2.3198	1.4156	0.6662
PSP (normalized to 0-1 range) cumulative area	-0.4103	0.3933	-1.2248	0.3285	0.2817
**Longitudinal process for PSP (normalized to 0-1 range)**					
Intercept	-2.3868	0.8871	-4.1896	-0.6933	0.0060
N_1(days)	0.3692	1.7737	-3.0551	3.8709	0.8426
N_2(days)	-0.8551	1.5791	-4.1242	2.1378	0.5767
Apache II	0.0464	0.0222	0.0029	0.0908	0.0369
CRP	0.0142	0.0338	-0.0524	0.0805	0.6643
σ	6.8608	0.9542	5.0099	8.7773	<0.0001

The terms N_i(days) denote the basis for a natural cubic spline with 2 degrees of freedom.
P, tail probability [2×min{P(·>0),P(·<0)}]; PSP, pancreatic stone protein; SD, standard deviation; VAP, ventilator-associated pneumonia.

Figure S1. The estimated cumulative incidence of VAP using the Kaplan-Meier approach and the competing risks approach.

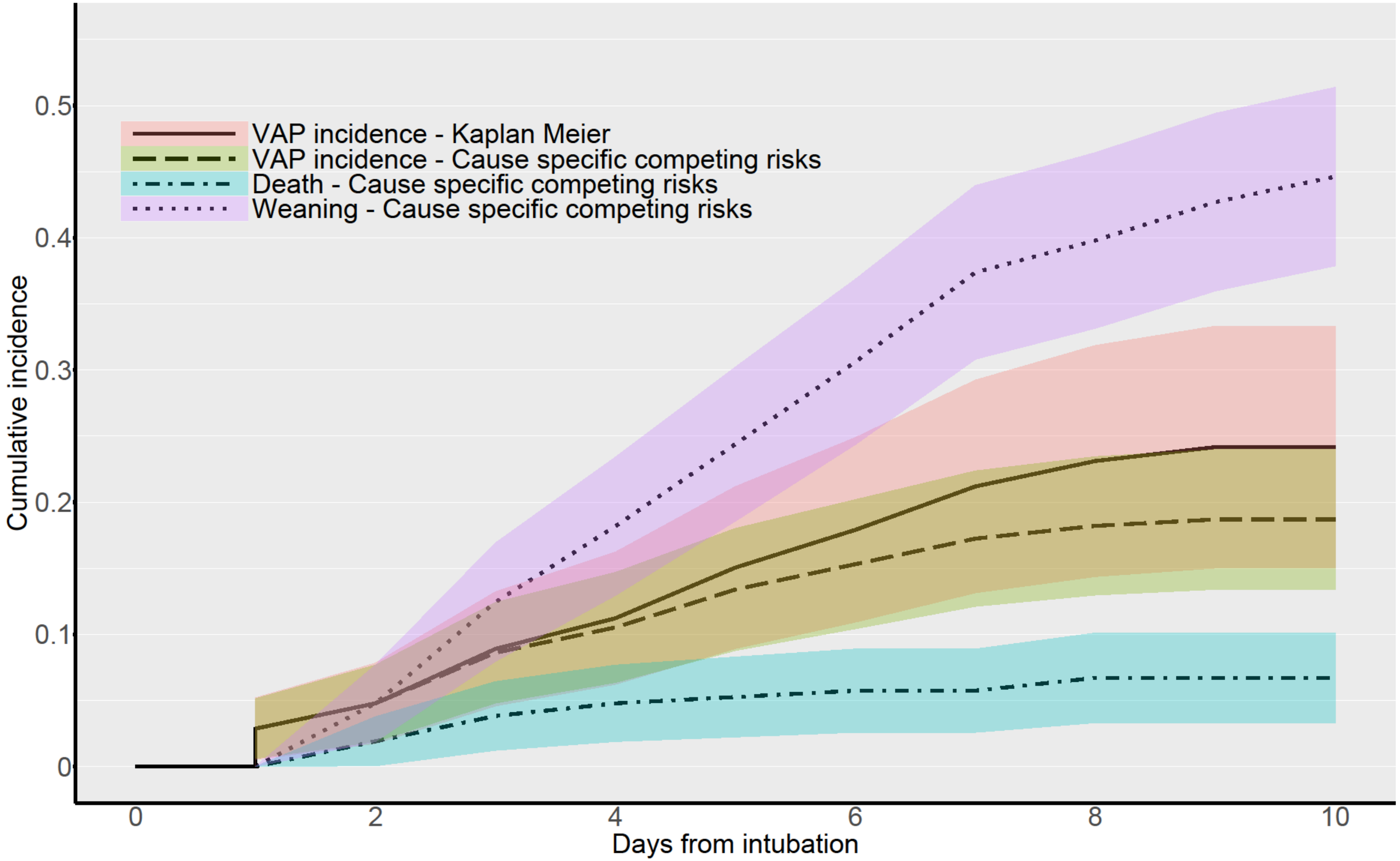

The solid line shows the cumulative incidence of VAP calculated using the Kaplan-Meier approach without accounting for competing risk events. The dashed line shows the cumulative incidence of VAP, after adjusting for competing risk. The dotted and dash-dotted lines show the cumulative incidence of weaning and death, respectively.

STROBE Statement—Checklist of items that should be included in reports of ***cohort studies***

	Item No	Recommendation	Page No
**Title and abstract**	1	(*a*) Indicate the study's design with a commonly used term in the title or the abstract	Title page
		(*b*) Provide in the abstract an informative and balanced summary of what was done and what was found	2
**Introduction**			
Background/rationale	2	Explain the scientific background and rationale for the investigation being reported	3
Objectives	3	State specific objectives, including any prespecified hypotheses	4
**Methods**			
Study design	4	Present key elements of study design early in the paper	4
Setting	5	Describe the setting, locations, and relevant dates, including periods of recruitment, exposure, follow-up, and data collection	4
Participants	6	(*a*) Give the eligibility criteria, and the sources and methods of selection of participants. Describe methods of follow-up	4-5
		(*b*) For matched studies, give matching criteria and number of exposed and unexposed	
Variables	7	Clearly define all outcomes, exposures, predictors, potential confounders, and effect modifiers. Give diagnostic criteria, if applicable	5-6

Data sources/ measurement	8*	For each variable of interest, give sources of data and details of methods of assessment (measurement). Describe comparability of assessment methods if there is more than one group	4-6
Bias	9	Describe any efforts to address potential sources of bias	5-6
Study size	10	Explain how the study size was arrived at	5-6
Quantitative variables	11	Explain how quantitative variables were handled in the analyses. If applicable, describe which groupings were chosen and why	6,7
Statistical methods	12	(*a*) Describe all statistical methods, including those used to control for confounding<br>(*b*) Describe any methods used to examine subgroups and interactions<br>(*c*) Explain how missing data were addressed<br>(*d*) If applicable, explain how loss to follow-up was addressed<br>(*e*) Describe any sensitivity analyses	6,7
**Results**			
Participants	13*	(a) Report numbers of individuals at each stage of study—eg numbers potentially eligible, examined for eligibility, confirmed eligible, included in the study, completing follow-up, and analysed<br>(b) Give reasons for non-participation at each stage<br>(c) Consider use of a flow diagram	8
Descriptive data	14*	(a) Give characteristics of study participants (eg demographic, clinical, social) and information on exposures and potential confounders	8, 9, 10. Table

		(b) Indicate number of participants with missing data for each variable of interest<br><br>(c) Summarise follow-up time (eg, average and total amount)	1. Fig 1
Outcome data	15*	Report numbers of outcome events or summary measures over time	8, 9

Main results	16	(*a*) Give unadjusted estimates and, if applicable, confounder-adjusted estimates and their precision (eg, 95% confidence interval). Make clear which confounders were adjusted for and why they were included<br><br>(*b*) Report category boundaries when continuous variables were categorized<br><br>(*c*) If relevant, consider translating estimates of relative risk into absolute risk for a meaningful time period	8-10<br><br>Table 2, Fig 2, 3, and supplementary
Other analyses	17	Report other analyses done—eg analyses of subgroups and interactions, and sensitivity analyses	Table 2, Fig 3
**Discussion**			
Key results	18	Summarise key results with reference to study objectives	10
Limitations	19	Discuss limitations of the study, taking into account sources of potential bias or imprecision. Discuss both direction and magnitude of any potential bias	13
Interpretation	20	Give a cautious overall interpretation of results considering objectives, limitations, multiplicity of analyses, results from similar studies, and other relevant evidence	10-14
Generalisability	21	Discuss the generalisability (external validity) of the study results	14
**Other information**			
Funding	22	Give the source of funding and the role of the funders for the present study and, if applicable, for the original study on which the present article is based	Title page

*Give information separately for exposed and unexposed groups.

Note: An Explanation and Elaboration article discusses each checklist item and gives methodological background and published examples of transparent reporting. The STROBE checklist is best used in conjunction with this article (freely available on the Web sites of PLoS Medicine at http://www.plosmedicine.org/, Annals of Internal Medicine at http://www.annals.org/, and Epidemiology at http://www.epidem.com/). Information on the STROBE Initiative is available at http://www.strobe-statement.org.
